# Headache As the Sole Symptom in Chronic Lymphocytic Inflammation With Pontine Perivascular Enhancement Responsive to Steroids (CLIPPERS): A Case Report and Literature Review

**DOI:** 10.7759/cureus.64310

**Published:** 2024-07-11

**Authors:** Omar Abdelkader, Hamza Abuzenah, Hans Shuhaiber

**Affiliations:** 1 Department of Neurology, Mayo Clinic, Jacksonville, USA; 2 Internal Medicine, Yarmouk University, Amman, JOR; 3 Department of Neurology, University of Florida College of Medicine, Gainesville, USA

**Keywords:** pontine, inflammatory brain disorder, steroids, headache, clippers

## Abstract

Chronic Lymphocytic Inflammation with Pontine Perivascular Enhancement Responsive to Steroids (CLIPPERS) is a rare central nervous system inflammatory condition usually presenting with a range of symptoms, including ataxia, diplopia, dysarthria, seizures, and headaches. We present a unique case of a 22-year-old woman exhibiting headache as the sole symptom. Imaging and biopsy confirmed the diagnosis, and initial steroid treatment provided relief, though it relapsed on tapering. Long-term management with low-dose steroids and mycophenolate mofetil achieved remission. This case highlights the importance of recognizing atypical presentations of CLIPPERS, emphasizing the need for prompt diagnosis and appropriate treatment plans to improve patient outcomes. Further research is necessary to enhance our understanding and management of CLIPPERS.

## Introduction

Chronic Lymphocytic Inflammation with Pontine Perivascular Enhancement Responsive to Steroids (CLIPPERS) is a rare inflammatory disorder of the central nervous system. It predominantly affects the pons but can also involve the midbrain and cerebellum. Clinical manifestations vary depending on the specific location of the pathology and can include ataxia, diplopia, dysarthria, and less commonly, seizures and headaches [1]. The diversity in clinical presentation can pose challenges in the diagnostic process. As such, adjunctive diagnostic modalities are often employed, such as brain MRI, typically revealing the characteristic “peppering of the pons” with gadolinium enhancement. Moreover, a biopsy with histopathological examination can reveal perivascular lymphocytic T-cell infiltration [2]. In this report, we present the case of a 22-year-old female patient with CLIPPERS who primarily exhibited severe headaches unaccompanied by other neurological symptoms. Prompt administration of steroids resulted in initial relief, yet subsequent steroid tapering led to a relapse. Further management involved the administration of low-dose steroids alongside mycophenolate, ultimately resulting in successful remission.

## Case presentation

**Figure 1:**
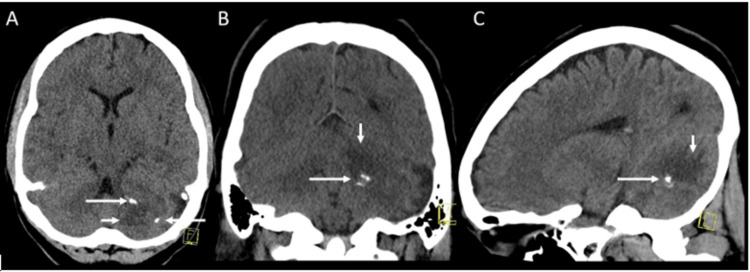
Non-enhanced CT of the head Axial (A), coronal (B), and sagittal (C) planes demonstrate subtle ill-defined hypodensity (short arrows) in the left cerebellum with scattered areas of punctate calcifications (long arrows), mainly along the margins of the lesion.

This is a case of a 22-year-old female with no prior medical or surgical history who presented with a primary complaint of headaches persisting for one month, often disturbing her sleep at night. Her family history is significant for a maternal diagnosis of neuromyelitis optica in the fourth decade of life. The initial physical exam was unremarkable, and the laboratory work included cerebrospinal fluid (CSF) analysis showing a normal panel with no oligoclonal bands and no malignancy on cytology, as well as negative results for myelin oligodendrocyte glycoprotein fluorescence-activated cell sorting (MOG FACS) and Neuromyelitis Optica (NMO)/AQP4 FACS autoantibodies. The patient mentioned a previous car accident, after which a computed tomography (CT) head scan revealed no abnormalities. However, the current CT imaging (Figure 1) identified a patchy, expansile, enhancing lesion in the left cerebellum extending into the middle cerebellar peduncle (MCP) with T2 changes.

**Figure 2:**
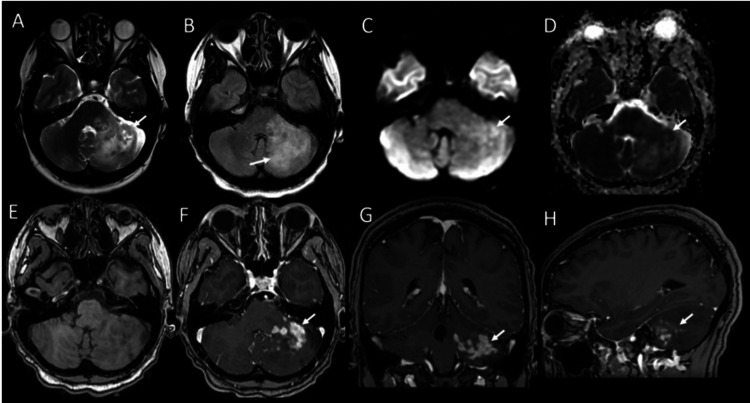
Contrast-enhanced MRI brain Axial T2 (A), FLAIR (B), DWI (C), ADC (D), pre-contrast 3DT1 (E), and postcontrast 3DT1 in axial (F), coronal (G) and sagittal (H) planes demonstrate multiple punctate, patchy, nodular, and linear lesions mainly involving the left middle cerebellar peduncle extending to left cerebellum and left aspect of the pons. Nodular T2 hypointense areas (arrow in A) with DWI hyperintensity (arrow in C) and corresponding ADC hypointensity (arrow in D) representing restricted diffusion compatible with a hypercellular process. There is surrounding mild edema in the left cerebellum and middle cerebellar peduncle on FLAIR (arrow in B); however, without significant mass effect. These lesions demonstrate prominent patchy, nodular, and punctate enhancement after administration of contrast (arrows in F, G, H) suggesting a lymphocytic perivascular inflammatory pattern. ADC: Apparent Diffusion Coefficient; DWI: Diffusion-Weighted Imaging; FLAIR: Fluid Attenuated Inversion Recovery; MRI: Magnetic Resonance Imaging

To narrow the differential, a Cosman-Roberts-Wells (CRW) biopsy with stealth MRI was recommended to guide further management. MRI (Figure 2) revealed a large, unusual, infiltrative, heterogeneously enhancing lesion in the left cerebellar hemisphere, extending into both the left MCP and pons. Imaging characteristics leaned more toward a neoplastic lesion than an inflammatory demyelinating lesion; however, histopathologic evaluation of the biopsy was needed to confirm the suspicion from imaging. While awaiting biopsy results, dexamethasone taper was initiated, starting from 2 mg BID and tapering down to 0.5 mg over four weeks.

**Figure 3:**
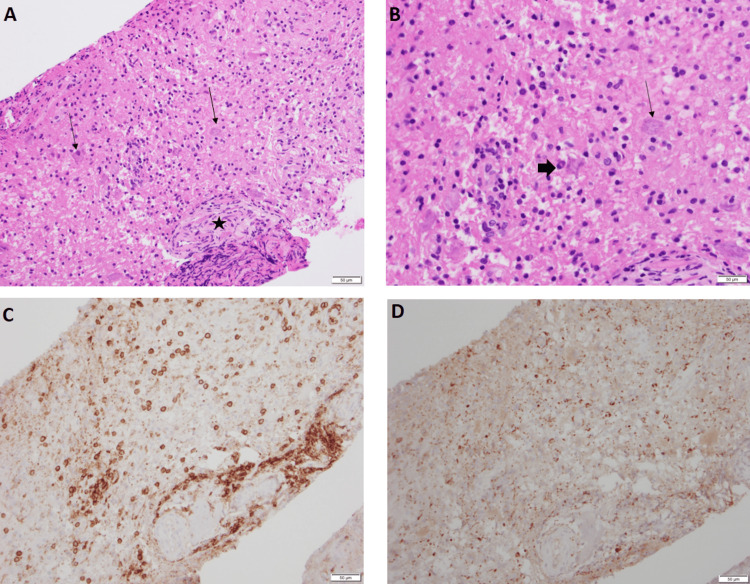
Histopathological findings using hematoxylin and eosin (H&E), CD4, and CD68 stains H&E stain 20× (A) and H&E stain 40×(B) sections show gray matter with prominent lymphoplasmacytic infiltrate. The arrowhead highlights plasma cells, arrows point to background native neurons. The star is a normal vessel. CD4 stain 20×(C) highlights T-lymphocytes with perivascular condensation and infiltration into adjacent tissue. CD68 20× stain(D) highlights microglia. Overall histology shows a chronic inflammatory process composed of a lymphoplasmacytic infiltrate with background reactive gliosis. There is no evidence of tumor in this material, and this is confirmed with negative next-generation sequencing as well as negative immunohistochemical stains for IDH1 and BRAF V600E.

Biopsy (Figure 3) revealed chronic inflammation and reactive changes, predominantly within cerebellar tissue displaying scattered CD4+ T cells and some CD20+ B cells. CD68 staining highlighted scattered macrophages and microglia. Immunohistochemical stains for IDH1 and BRAF V600E mutations, as well as for JC virus and toxoplasmosis, were negative. The NeuN immunohistochemical stain highlighted native neurons without dysmorphology, and focal Rosenthal fibers were identified, indicating reactive gliosis. GlioSeq molecular testing revealed no alterations suggestive of a neoplastic process. Contrary to the preliminary imaging-based diagnosis, the biopsy findings favored an inflammatory, non-infectious, and non-neoplastic process.

Following imaging and biopsy assessment, the differential diagnosis was narrowed to CLIPPERS versus another inflammatory condition of the brain. The patient reported improvement in headache symptoms during the glucocorticoid taper. Consequently, the next steps included a neurological evaluation and a plan to restart steroids if her symptoms recurred. A week after the completion of the steroid taper, the patient reported the return of severe headaches that again disturbed her sleep and caused daytime drowsiness. Notably, she did not experience symptoms of nausea, vomiting, or changes in vision, and neurological evaluation revealed no focal deficits. Dysmetria was not observed in the finger-to-nose test, and motor strength was 5/5 in both the upper and lower extremities.

Based on previous MRI and biopsy results, along with the patient’s response to steroids, CLIPPERS was determined to be the appropriate diagnosis. Prednisone 20 mg QD was started along with steroid-sparing mycophenolate mofetil 500 mg BID. Treatment commenced with prednisone 20 mg once daily complemented by steroid-sparing mycophenolate mofetil 500 mg twice daily. Additionally, the patient was initiated on topiramate 25 mg BID, and rizatriptan 5 mg for use at headache onset, with dietary adjustments recommended to mitigate headache symptoms by reducing the intake of sugary foods. The vitamin D level was low at 18.75 ng/ml, prompting the prescription of vitamin D supplements to alleviate symptoms of weakness and fatigue. A complete blood count and comprehensive metabolic panel were ordered to investigate other potential underlying causes of the new-onset weakness. A follow-up plan was established for a one-month interval to repeat the MRI, monitor headache progression, and evaluate for the appearance of new symptoms such as ataxia, dysarthria, and diplopia.

## Discussion

CLIPPERS is a rare condition first reported by Pittock et al. in 2010 [3]. While this condition can affect individuals of all ages and both sexes, Al-Chalabi et al. conducted a systemic review in 2022 encompassing 140 CLIPPERS patients, and reported a mean age of 46.5 years, with a male predominance of 75% [4]. This designates our 22-year-old female patient as a unique case concerning both age and sex, underscoring the necessity of considering CLIPPERS in patients of all age groups and genders. As a relatively recent condition, the pathogenesis remains largely obscure, nonetheless, multiple hypotheses have been postulated including organ-specific autoimmunity, the impact of viral infections on autoimmunity, and Th17-mediated immunity [5]. Concurrently, a gene variation library for CLIPPERS was established, offering potential insights into the etiology and pathogenesis of the condition [6].

Primary lesions predominantly affect the pons, although CLIPPERS pathology can also extend to other regions of the brainstem, corpus callosum, white matter of the cerebellum, and basal ganglia, as well as the spinal cord. These lesions manifest on enhanced MRI brain scans as thin curvilinear high signal enhancement shadows, resembling pepper-like enhancement [1]. Clinical presentations exhibit variability, as illustrated by the diverse array of symptoms observed among the 140 patients in Al-Chalabi et al. systematic review. The most prevalent manifestations included ataxia (92.8%), diplopia (51.4%), and dysarthria (50.0%), while cognitive impairment (17.0%), seizures (3.5%), and headache (1.4%) were less frequently reported [4].

Headache, although often overlooked, has been reported in five cases to date. Initially noted by Simon et al. in 2012 [7], followed by subsequent reports by Buttman et al. in 2013 [8], Cordano et al. 2018 [9], and Mubasher et al. in 2017 [10], and most recently in a pediatric case by Lee et al. in 2022 [11]. Notably, in these cases, headache consistently coexisted with other symptoms of CLIPPERS such as ataxia, dysarthria, diplopia, and numbness. Whereas headache as the sole presenting symptom as observed in our case has not been previously reported.

Diagnostic criteria for CLIPPERS were first established by Simon et al. in 2012 [7], and a more comprehensive framework was outlined incorporating clinical, MRI, and neuropathologic criteria. A definitive diagnosis of CLIPPERS is concluded if a patient fulfills the three criteria, while a probable diagnosis is made if they fulfill the clinical and radiological criteria only [12], CLIPPERS is frequently underestimated and presents a diagnostic challenge due to its broad spectrum of mimicking conditions and variable clinical presentations. In consequence, delays in diagnosis and treatment frequently occur, resulting in poorer patient outcomes [4]. Several conditions mimicking CLIPPERS have been identified, including myelin oligodendrocyte glycoprotein antibody disease (MOGAD), and neuromyelitis optica (NMO). Ferilli et al. recently reported a case presenting with a headache responsive to steroids, along with MRI findings of patchy lesions in the pons, left MCP, and medulla with post-gadolinium enhancement. These clinical and radiological features suggested a “probable” diagnosis of CLIIPERS. Nevertheless, the presence of optic neuritis and conus medullaris lesions argued against the diagnosis of CLIPPERS, warranting further investigations for MOGAD which ultimately confirmed a diagnosis of MOGAD mimicking CLIPPERS syndrome [13]. Considering our patient’s family history of NMO, this observation may lend support to the hypothesis of organ-specific autoimmunity in CLIPPERS pathogenesis, and further studies are likely warranted to elucidate a potential association between NMO and CLIPPERS.

Regardless of concerns regarding the inherent risks and potential complications associated with biopsy due to the involvement of critical brain regions, as well as its impracticality in certain cases [2], there exists a prevalent argument on the imperative nature of biopsy [7], particularly in patients with atypical presentations [14]. Pessoa et al. emphasized the necessity of performing the biopsy before corticosteroid administration to prevent false negative results [15]. Histological analysis, consistent with our case, should exhibit perivascular lymphocytic T-cell infiltration with an increased CD4 to CD8 ratio [2,16]. Al-Chalabi et al. reported that 55 % out of the 140 CLPPERS patients underwent brain biopsy [4], whereas Pittock et al. reported that 50 % of their patients were successfully diagnosed and treated without biopsy [3].

Corticosteroids are fundamental in the acute management of CLIPPERS, eliciting both clinical and radiological responses [1,7]. Al-Chalabi et al. underscored that the duration of steroid therapy in the acute phase was significantly shorter in the relapsed group compared to the non-relapsed group (mean 6.19±7.9 vs. 10.14±12.1 days, respectively, P = 0.04) [4], reinforcing the recommendation to administer high-dose steroids for 10 days in the acute phase, a directive also endorsed by Taieb et al. [17].

Relapse is deemed the most prevalent complication associated with CLIPPERS, often correlated with rapid steroid tapering, and leading to worsened outcomes and increased mortality rates [4,16]. In our case, a recurrence of severe headaches was observed one week after steroid tapering, despite a 15-day course of dexamethasone. This could potentially be attributed to the prolonged symptomatic period of one month with a severe headache before treatment.

Despite Alexerad et al. concerns regarding the adverse effects of chronic steroid treatment [2], there is a widespread consensus that such treatment is essential. This agreement stems from the recognition that attempts to taper glucocorticoids almost inevitably lead to neurological relapse, even when a steroid-sparing agent is administered in isolation, without concurrent corticosteroids [1,4,7].

Given the limitations and adverse events posed by chronic steroid treatment, various steroid-sparing immunosuppressants have been employed to sustain clinical improvement and prevent relapse [1]. Al-Chalabi et al. reported that among the 140 CLIPPERS patients, 69% utilized steroid-sparing agents, with azathioprine being the most frequently used (35%), followed by methotrexate (34%), mycophenolate (15%), and cyclophosphamide (13%) [4]. In our case, for long-term management, we administered a regimen compromising low-dose prednisolone alongside mycophenolate, a treatment also employed by Cornado in his patient presenting with headaches [9].

In conclusion, CLIPPERS remains a challenging and underdiagnosed condition with various clinical presentations. Our case of a 22-year-old female patient with an atypical presentation of CLIPPERS, primarily presenting with severe headache without typical neurological symptoms, emphasizes the importance of recognizing the broad spectrum of clinical presentations associated with this condition. This also highlights the significance of neurologists and headache specialists maintaining a high index of suspicion for CLIPPERS in their practice, as timely recognition and treatment are crucial for favorable outcomes.

Prompt initiation of steroid therapy is fundamental for acute management and given the high risk of relapse often linked to rapid steroid tapering, cautious management and consideration of steroid-sparing immunosuppressant agents are warranted. As our understanding of CLIPPERS continues to evolve, further research is warranted to elucidate its pathophysiology, refine diagnostic criteria, and optimize treatment strategies.

## Conclusions

In conclusion, CLIPPERS remains a challenging and underdiagnosed condition with various clinical presentations. Our case of a 22-year-old female patient with an atypical presentation of CLIPPERS, primarily presenting with severe headache without typical neurological symptoms, emphasizes the importance of recognizing the broad spectrum of clinical presentations associated with this condition. This also highlights the significance of neurologists and headache specialists maintaining a high index of suspicion for CLIPPERS in their practice, as timely recognition and treatment are crucial for favorable outcomes.

Prompt initiation of steroid therapy is fundamental for acute management and given the high risk of relapse often linked to rapid steroid tapering, cautious management and consideration of steroid-sparing immunosuppressant agents are warranted. As our understanding of CLIPPERS continues to evolve, further research is warranted to elucidate its pathophysiology, refine diagnostic criteria, and optimize treatment strategies.
